# Co-expression of GAD67 and choline acetyltransferase in neurons in the mouse spinal cord: A focus on lamina X

**DOI:** 10.1016/j.brainres.2016.07.001

**Published:** 2016-09-01

**Authors:** Jittima Gotts, Lucy Atkinson, Yuchio Yanagawa, Jim Deuchars, Susan A. Deuchars

**Affiliations:** a School of Biomedical Sciences, Faculty of Biological Sciences, University of Leeds, Leeds LS2 9JT, United Kingdom; b Department of Genetic and Behavioural Neuroscience, Gunma University Graduate School of Medicine, 3-39-22 Showa-machi, Maebashi 371-8511, Japan

**Keywords:** Acetylcholine, GABA, Spinal cord, Interneuron, Central canal, Lamina X

## Abstract

Lamina X of the spinal cord is a functionally diverse area with roles in locomotion, autonomic control and processing of mechano and nociceptive information. It is also a neurochemically diverse region. However, the different populations of cells in lamina X remain to be fully characterised. To determine the co-localisation of the enzymes responsible for the production of GABA and acetylcholine (which play major roles in the spinal cord) in lamina X of the adult and juvenile mouse, we used a transgenic mouse expressing green fluorescent protein (GFP) in glutamate decarboxylase 67 (GAD67) neurons, combined with choline acetyltransferase (ChAT) immunohistochemistry. ChAT-immunoreactive (IR) and GAD67-GFP containing neurons were observed in lamina X of both adult and juvenile mice and in both age groups a population of cells containing both ChAT-IR and GAD67-GFP were observed in lumbar, thoracic and cervical spinal cord. Such dual labelled cells were predominantly located ventral to the central canal. Immunohistochemistry for vesicular acetylcholine transporter (VAChT) and GAD67 revealed a small number of double labelled terminals located lateral, dorsolateral and ventrolateral to the central canal. This study therefore describes in detail a population of ChAT-IR/GAD67-GFP neurons predominantly ventral to the central canal of the cervical, thoracic and lumbar spinal cord of adult and juvenile mice. These cells potentially correspond to a sub-population of the cholinergic central canal cluster cells which may play a unique role in controlling spinal cord circuitry.

## Highlights

• GABA and ACh synthesising enzymes are co-localised in the mouse spinal cord.
• Such cells are predominantly located ventral to the central canal in lamina X.
• These cells may be a subpopulation of cholinergic central canal cluster neurons.

## 1 Introduction

The neurotransmitters GABA and acetylcholine (ACh) play important functional roles in the spinal cord. GABAergic inhibitory interneurons are critical components of local circuits and are abundant in the dorsal horn and ventral horn where they modulate sensory processing and motor activity (Todd and Maxwell, 2000). Neurons containing immunoreactivity for GABA or the synthesising enzyme glutamate decarboxylase (GAD) are found in the dorsal horn of the rat and mouse (Kaduri et al., 1987, Polgar et al., 2013, Todd and McKenzie, 1989) with scattered somata in other layers, particularly lamina X and cerebrospinal fluid contacting neurons in the ependymal layer surrounding the central canal (Kaduri et al., 1987). The cholinergic system is also important in the spinal cord, since ACh is an important modulator of sensory processing and motor output at the spinal cord level. The ACh synthesising enzyme choline acetyltransferase (ChAT) is expressed by ventral horn motoneurons, small cells clustered around the central canal, partition neurons in the intermediate grey area, preganglionic sympathetic and parasympathetic neurons and neurons in the dorsal horn of the adult rat (Barber et al., 1984).

One study suggested the possibility of a role for cells expressing both ACh and GABA in lamina X in the cervical spinal cord of the rat (Kosaka et al., 1988), but did not examine other spinal levels that are also critical for locomotion and autonomic control. Lamina X is an important site for the convergence of somatic and visceral afferent inputs conveying mechanoreceptive and nociceptive information (Honda, 1985, Honda and Lee, 1985, Honda and Perl, 1985, Nahin et al., 1983, Ness and Gebhart, 1987). This area also contains neuronal networks responsible for the generation of locomotor behaviours (Bertrand and Cazalets, 2011), including cholinergic neurons (Zagoraiou et al., 2009). In addition, there are inhibitory neurons in lamina X that project to sympathetic preganglionic neurons of the intermediolateral cell column (Deuchars et al., 2005). Since lamina X also contains sympathetic preganglionic neurons (Deuchars and Lall, 2015), it is a neurochemically and functionally diverse region.

Understanding the neurochemical diversity of cells in lamina X may therefore contribute to unravelling the function of this region. Since each level of the spinal cord may contain unique circuitry underlying specific functions, lamina X may vary in cellular composition and structure throughout the cord. Further, potential species differences in cell phenotypes should be considered. Therefore, since mouse is a commonly utilised experimental model we have examined the distribution of double labelled cholinergic and GABAergic cells in lamina X of the spinal cord. Since previous studies have focussed on the cervical spinal levels, we extend this to lumbar and thoracic cord. To enhance detection of GAD expressing neurones, we utilised a transgenic mouse in which GAD67 is reported by expression of GFP in conjunction with immunohistochemistry for ChAT. Since understanding the localisation could be a precursor to electrophysiological studies and juvenile mice are frequently used for such studies *in vitro*, we examined the distribution in both juvenile and adult mice. To investigate where the double labelled cells could project, the localisation of vesicular acetylcholine transporter (VAChT) and GAD67 labelled terminals in the spinal cord were examined.

## 2 Results

### 2.1 Verification of GAD67-GFP knock-in mice

**Fig. 1:**
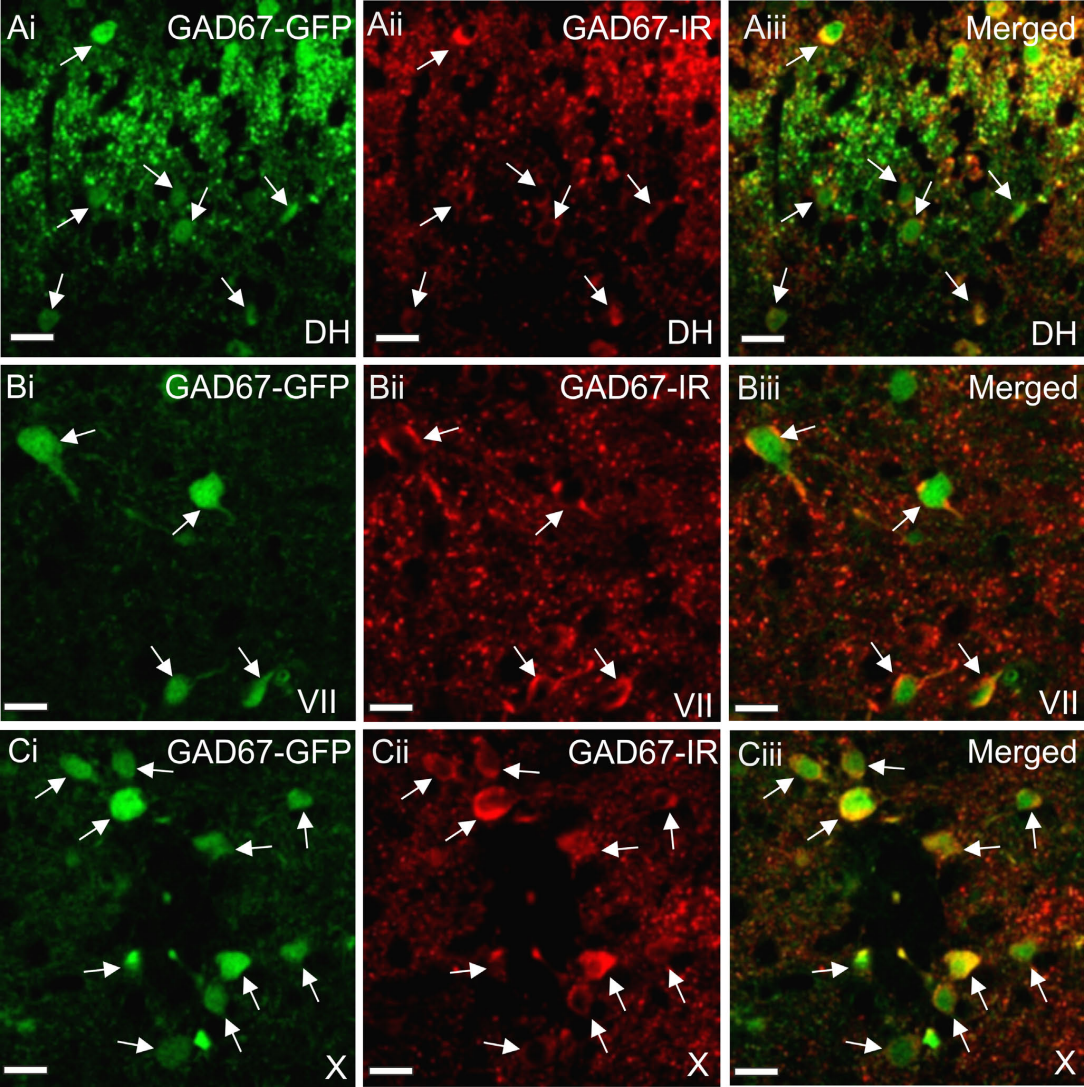
*Distribution of GAD67-GFP and GAD67-IR cells in the adult mouse spinal cord*. Confocal images showing GAD67-GFP containing cells in the dorsal horn (Ai), lamina VII (Bi) and Lamina X (Ci). GAD67-IR cells are also observed in these areas (Aii, Bii, Cii) and merging of the images reveal that GAD67-GFP and GAD67-IR is observed in the same cells, arrows indicate double labelled cells. Scale bars=20 µm.

To verify that the GFP labelled cells in this model contain GAD67 we performed immunohistochemistry for GAD67 on spinal cord sections from GAD67-GFP mice. Co-localisation of GAD67-GFP and GAD67-IR was observed in cells throughout the spinal grey matter including the dorsal horn (Fig. 1Ai-iii), lamina Vii (Fig. 1Bi-iii) and lamina X surrounding the central canal (Fig. 1Ci-iii). The distribution of these two markers for GAD67 was examined more closely in lamina X, where 100% of GAD67-IR lamina X neurons (in the cervical, thoracic and lumbar spinal cord) also contained GAD67-GFP, indicating that the knock-in mice express GFP in GAD67-IR cells. In addition, 76% (cervical), 85% (thoracic) and 86% (lumbar) of GAD67-GFP cells contained GAD67-IR. Since GFP expression is more reliably detected and more visible in cell somata than GAD67, the GAD67-GFP knock in mouse constitutes a suitable model to observe labelled cell bodies of GAD67 containing cells in the spinal cord.

### 2.2 Distribution of GAD67-GFP and ChAT-IR in the spinal cord of adult mice

**Fig. 2:**
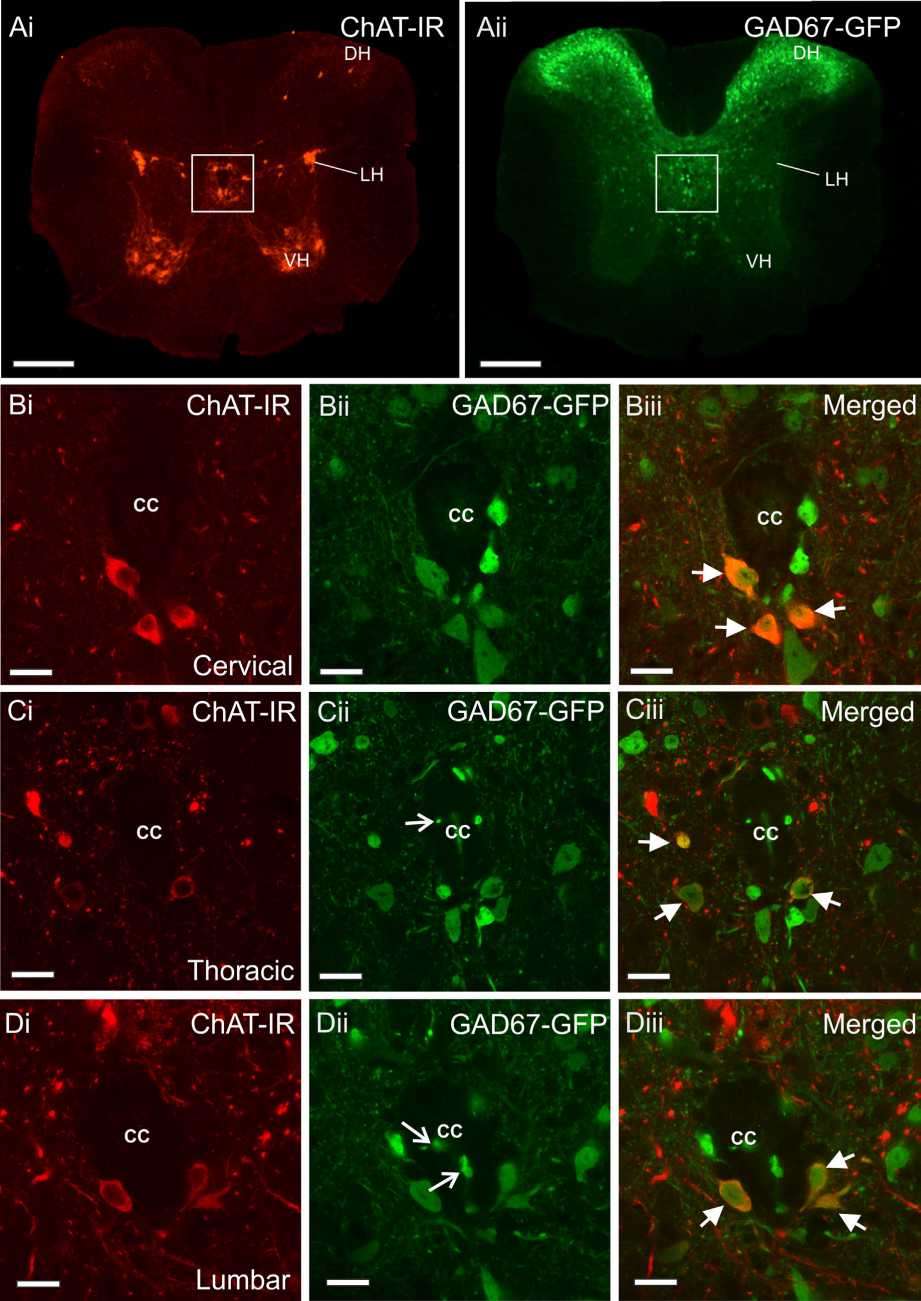
*Distribution of ChAT-IR and GAD67-GFP cells in the adult mouse spinal cord, with a focus on lamina X.* Ai and Aii) Low power fluorescent images showing the distribution of ChAT-IR (Ai) and GAD67-GFP (Aii) cells in the spinal cord. ChAT-IR cells are observed in the ventral horn (VH), lateral horn (LH), dorsal horn (DH) and lamina X surrounding the central canal (boxed area). GAD67-GFP labelled cells were observed predominantly in the dorsal horn and yet also distributed around the central canal in lamina X, and a few were observed in the lateral horn and lamina VII. Scale bars=200 µm B–D) Confocal images showing ChAT-IR neurons surrounding the central canal (cc) in the cervical (Bii), thoracic (Cii) and lumbar (Dii) spinal cord. GAD67-GFP containing neurons are also present in these areas (Bii, Cii and Dii). GAD67-GFP containing neurons are observed in the cell bodies of cerebrospinal fluid contacting neurons and their terminal bulb like structures which they send into the central canal (open arrows show labelled bulb like structures in Cii and Dii). In all levels of the spinal cord studied double labelled cells containing both ChAT-IR and GAD67-GFP can be observed (Biii, Ciii and Diii, closed arrows point to double labelled neurons). These are predominantly located ventral and occasionally ventrolateral to the central canal. Scale bars=50 µm.

ChAT-IR and GAD67-GFP labelled cells were observed in many areas of the spinal cord (Fig. 2Ai and Aii), both sets of cells were oval or elliptical in shape and yet varied in size. The mean numbers of ChAT-IR, GAD67-GFP and dual labelled ChAT-IR/GAD67-GFP cells in each area at each level of the spinal cord studied are shown in Table 1.

#### 2.2.1 ChAT-IR and GAD67-GFP cells in lamina X of adult mice

The focus of this study is on the distribution of ChAT-IR and GAD67-GFP neurons in lamina X of the spinal cord. Single labelled ChAT or GAD67-GFP containing neurons were observed in this area in the cervical, thoracic and lumbar regions of the spinal cord. ChAT-IR neurons were distributed throughout lamina X (Fig. 2Bi, Ci and Di) and on average, the dimensions of their cell somata were 13.62±0.95 µm×19.52±1.68 µm (N=10), however these varied greatly, ranging from 8 µm to 27 µm for the X-axis and from 9 µm to 47 µm for the Y-axis of the cell body (N=26). These may correspond to the larger central autonomic ChAT containing cells and smaller central cluster neurons as described by Barber et al. (1991).

GAD67-GFP neurons in lamina X were observed surrounding the central canal with a morphology indicative of cerebrospinal fluid contacting neurons (CSFCNs) (*i.e.* located close to the ependymal cell layer and having a projection ending in a bulb like structure in the central canal (Fig. 2Cii and Dii)). In addition, some GAD67-GFP neurons were close to the ependymal cell layer with no bulb like projections into the central canal (Fig. 2Bii, Cii and Dii). On average the size of GAD67-GFP neurons in lamina X was 11.14±0.36 µm×15.08±0.57 µm (N=10).

**Fig. 3:**
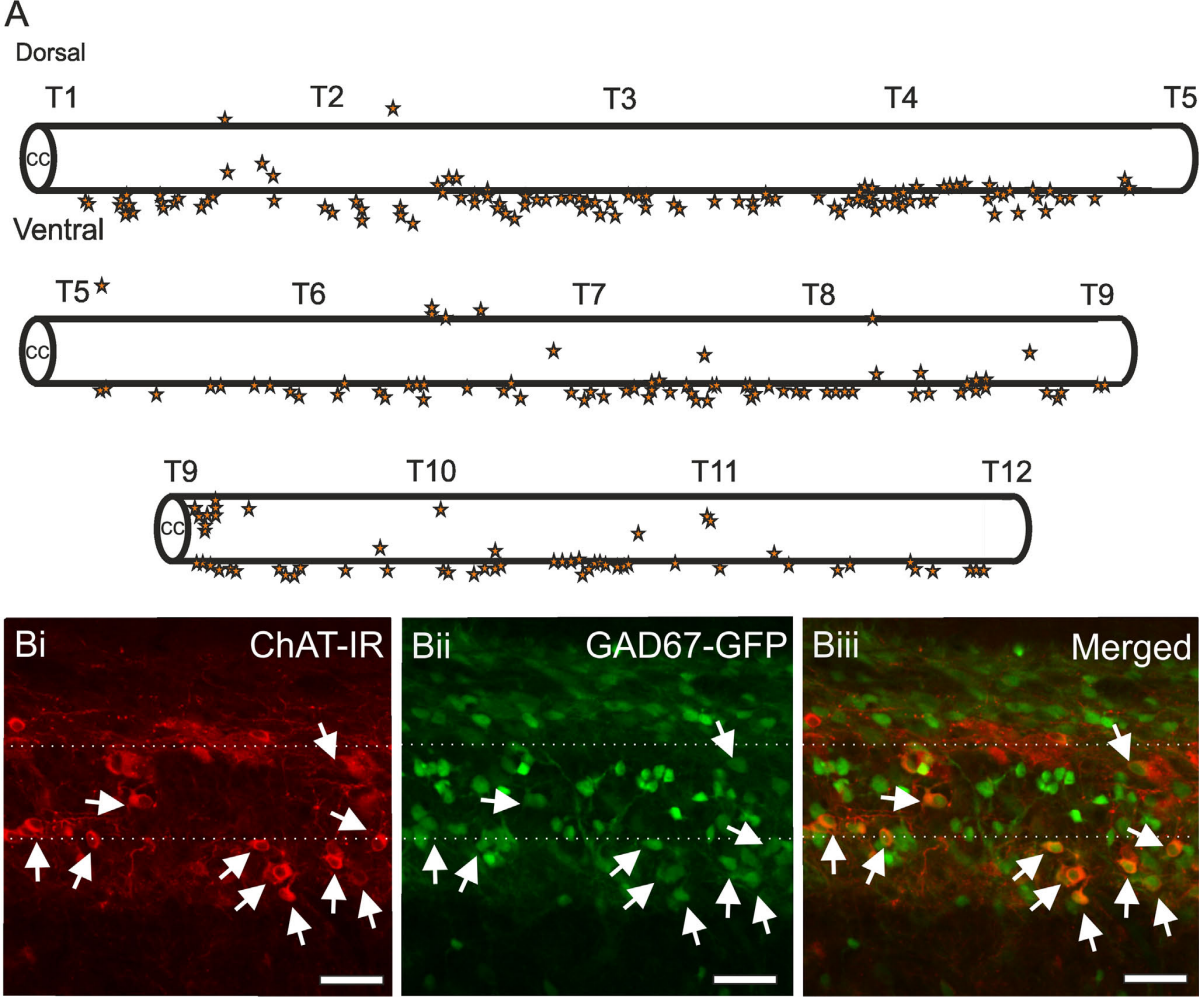
*Double labelled ChAT-IR/GAD67-GFP labelled cells in lamina X are located predominantly ventral to the central canal*. A. Schematic representation of a longitudinal section of the thoracic spinal cord (T1–T12). Double labelled ChAT-IR/GAD67-GFP cells are represented by a star and are predominantly located ventral and ventrolateral to the central canal with a small minority located dorsal to the central canal. Bi-Biii) Confocal images of a longitudinal section of the thoracic spinal cord showing the distribution of ChAT-IR (Bi) and GAD67-GFP labelled cells (Bii) along the central canal. Double labelled ChAT-IR and GAD67-GFP cells can be observed in the merged image (Biii, arrows) predominantly ventral to the central canal. Dotted lines represent the approximate borders of the central canal. Scale bars=50 µm.

Dual labelled ChAT-IR/GAD67-GFP neurons in lamina X had somata measuring 11.35±0.66 µm×14.68±0.55 µm (N=10) and appeared lightly immunoreactive for both GAD67-GFP and ChAT (Fig. 2Biii, Ciii, Diii). These dual labelled ChAT-IR/GAD67-GFP cells were not CSFCNs as determined by the absence of a projection into the central canal. Mapping of these double labelled neurons revealed they were located predominantly ventral and ventrolateral to the central canal, which was particularly apparent in longitudinal sections of the spinal cord, however a smaller number were observed dorsal to the central canal (Fig. 3A and Bi-iii).

Lamina X contains the majority of dual labelled ChAT-IR/GAD67-GFP cells in the spinal cord. The percentages of ChAT-IR neurons that also contain GAD67-GFP are 37% at the cervical level, 27% at the thoracic level and 33% at the lumbar level (see Table 1).

### 2.3 The distribution of VAChT and GAD67-IR terminals in the adult mouse spinal cord

**Fig. 4:**
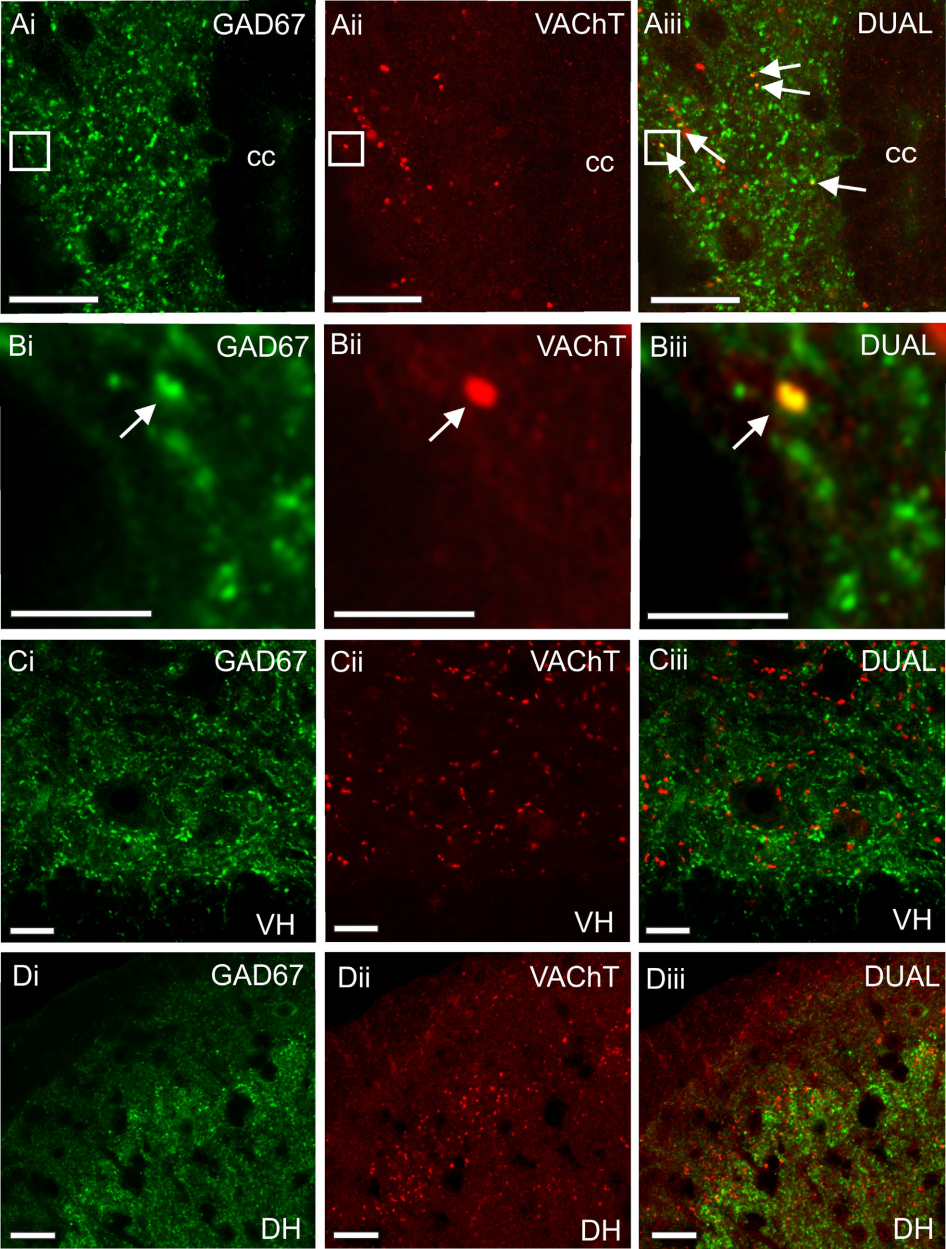
*VACHT-IR and GAD67-IR terminals surrounding the central canal*. Confocal images showing VAChT-IR and GAD67-IR terminals in the spinal cord. A and B) GAD67-IR terminals (Ai and Bi) and VAChT-IR terminals (Aii and Bii) are observed surrounding the central canal (CC). VAChT-IR terminals that also contain GAD67-IR are observed lateral to the central canal (Aiii and Biii). Boxed area in A shown at higher power in B. C) GAD67-IR (Ci) and VAChT-IR (Cii) terminals are observed in the ventral horn yet do not colocalise in this area (Ciii) D) GAD67-IR (Di) and VAChT-IR (Dii) terminals are observed in the dorsal horn yet do not colocalise in this area (Diii) Scale bars in A, C and D=20 µm and in B=5 µm.

To determine the possible termination sites of the double labelled ChAT/GAD67-IR cells, we performed immunohistochemistry for the vesicular acetylcholine transporter (VAChT) and GAD67. VAChT immunoreactivity was observed prominently within the motoneuron cell groups of the ventral horn in large terminals (putative C terminals, Fig. 4Cii). In addition, smaller VAChT-IR terminals were throughout the grey matter, including surrounding the central canal in lamina X and the dorsal horn. This distribution was similar to that previously described in the rat (Ranson et al., 2007, Schafer et al., 1998). GAD67-IR terminals that were also VAChT-IR were a very small population that exclusively surrounded the central canal in lamina X. These double labelled terminals were never onto the ependymal cell layer itself but always a short distance away from the central canal and located dorsolateral, lateral (Fig. 4A and B) and ventrolateral to the central canal. No double labelled GAD67-IR/VAChT-IR terminals were observed in the ventral horn (Fig. 4Ci-Ciii) or the dorsal horn (Fig. 4Di-Diii).

### 2.4 Distribution of GAD67-GFP and ChAT-IR in spinal cord lamina X of juvenile mice

As juvenile mice are more commonly utilised than adult mice for electrophysiological experiments due to lower levels of myelination, we also determined the distribution of ChAT-IR and GAD67-GFP containing cells in lamina X of juvenile mice.

**Fig. 5:**
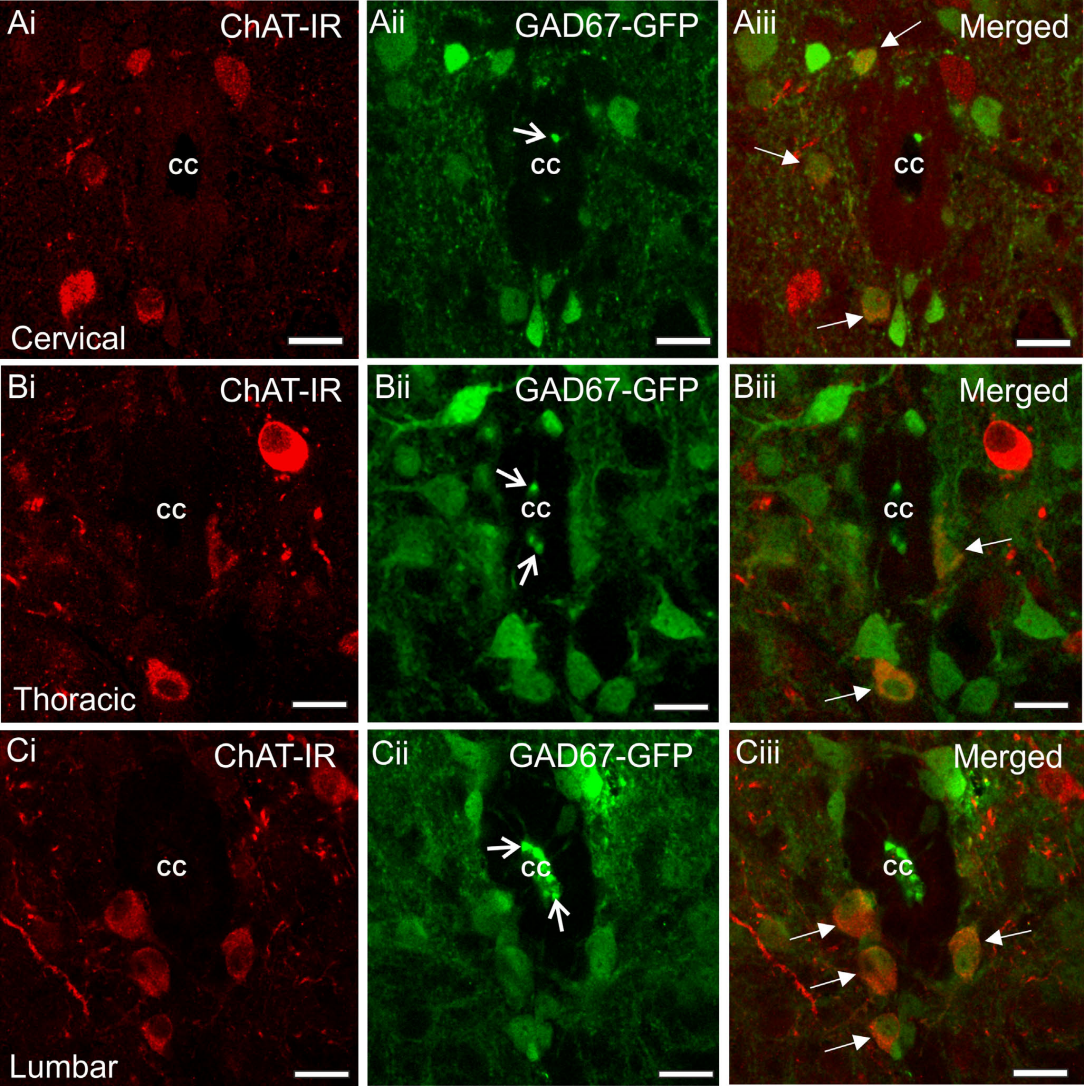
*Distribution of ChAT-IR and GAD67-GFP cells in lamina X of the juvenile mouse*. Confocal images showing ChAT-IR neurons are present surrounding the central canal (cc) in the cervical Ai), thoracic (Bi) and lumbar (Ci) spinal cord. GAD67-GFP containing neurons are also present in these areas (Aii, Bii and Cii). GAD67-GFP is observed in the cell bodies of cerebrospinal fluid contacting neurons and their terminal bulb like structures which they send into the central canal (open arrows show labelled bulb like structures in Aii, Bii and Cii). In all levels of the spinal cord studied double labelled cells containing both ChAT-IR and GAD67-GFP can be observed (Aiii, Biii and Ciii). These are predominantly located ventral and occasionally ventrolateral to the central canal. Arrows indicate double labelled neurons. Scale bars=20 µm.

ChAT-IR neurons (16.17±0.57 µm×21.60±1.13 µm, N=10) and GAD67-GFP containing neurons (13.21±0.63 µm×16.17±0.98 µm, N=10, Fig. 5) were found in lamina X of cervical, thoracic and lumbar sections. Similar to that observed in adult mice, some GAD67-GFP neurones had the morphology and distribution of CSFCNs whilst others were located subependymally and did not contain bulb-like projections into the central canal and so were not CSFCNs.

Similar to adult mice, double labelled ChAT-IR/ GAD67-GFP containing neurons were high in number in lamina X of juvenile mice at all spinal levels (Fig. 5Aiii, Biii and Ciii). These measured 12.68±0.47 µm×16.90±1.11 µm (N=10) and appeared lightly immunoreactive for both GAD67-GFP and ChAT. The percentage of ChAT-IR neurons in this area that also contained GAD67-GFP was 41% in the cervical level, 19% in the thoracic level and 18% in the lumbar level. No double labelled ChAT-IR/GAD67 GFP labelled cells had projections into the central canal suggesting they were not CSFCNs.

## 3 Discussion

This study provides a detailed description of the distribution of double labelled ChAT-IR/GAD67-GFP containing cells in lamina X of the spinal cord. This confirms that the GABA/ChAT cells reported in rat cervical spinal cord (Kosaka et al., 1988) are present in the mouse, but also extends knowledge to reveal them also in the thoracic and lumbar spinal cord. Further, the distribution of the cells is revealed as similar in adult and juvenile mice. Potential projections of these neurons were identified through co-localisation of vesicular acetylcholine transporter (VAChT) and GAD67 labelled terminals. This description of these cells in the mouse and localisation in the thoracic and lumbar spinal cord adds to our knowledge on the neurochemical diversity of lamina X in the spinal cord.

### 3.1 Technical considerations

Two isoforms of the GABA synthesising enzyme glutamic acid decarboxylase (GAD) exist: GAD65 and GAD67. The distributions of GAD65 and GAD67-IR cell bodies are similar in the spinal cord of the rat (Feldblum et al., 1995) and cat (Tillakaratne et al., 2000), with both forms present throughout the dorsal horn and lamina X, whereas GAD67 is the predominant form in the ventral horn. Potential co-expression of the two isoforms is supported by examination of GAD-IR axonal boutons with antibodies to GAD65 and GAD67 in the rat spinal cord, where all terminals were labelled with both antibodies (Mackie et al., 2003). Therefore, the presence of GAD67 seems likely to reveal the majority of GABAergic neurons in the spinal cord.

Since localisation of GAD or GABA with antibodies has proven unreliable in the spinal cord and brainstem, transgenic mice expressing reporters such as GFP in GABAergic neurons have previously been used to examine GABAergic cells in the CNS. In GAD67-GFP mice created by (Oliva et al., 2000), 68% of lamina I GABAergic neurons at P14 (Dougherty et al., 2005) and 35% of lamina II GABAergic neurons in the adult (Heinke et al., 2004) expressed GFP. It has since become clear that in many CNS regions this mouse line expresses GFP only in a subset of GABAergic neurons, probably because GFP expression is controlled by a relatively short segment of the GAD67 promoter (Ma et al., 2006). In this study we utilised a GAD67-GFP knock-in mouse which expresses GFP under control of the endogenous GAD67 promoter (Tamamaki et al., 2003). This mouse is widely used to study the structure and functions associated with the GABAergic system in the CNS (Brown et al., 2008, Gotts et al., 2015, Okada et al., 2008, Ono et al., 2005). Further, we verified that this line is a suitable model for study of lamina X since 100% of GAD67-IR neurons in this region also contained GAD67-GFP. The observation that not all GAD67-GFP cells contain GAD67-IR is likely due to the difficulty of using immunohistochemistry for GAD67 to obtain exact numbers of labelled GAD67-IR cell bodies (Fong et al., 2005) and the more limited tissue penetration of antibodies to GAD67.

### 3.2 Lamina X ChAT-IR/GAD67-GFP cells in the adult and juvenile mouse

Small populations of neurons in lamina X of the spinal cord containing the synthesising enzymes for both acetylcholine and GABA were found in both adult and juvenile mice. The size and location of the double labelled cells ventral to the central canal suggests they could correspond to the ChAT-IR ventral “central canal cluster neurons” which are small peri-ependymal neurons with thin short dendrites (Barber et al., 1984; Phelps et al., 1984). These may include the propriospinal ChAT-IR cells located close to the central canal (often ventrally) which were labelled by injection of a retrograde tracer into the grey matter of the third lumbar spinal segment of the rat (Sherriff and Henderson, 1994).

Lamina X cholinergic central canal cluster neurons are involved in locomotion (Bertrand and Cazalets, 2011). Central cluster cholinergic cells are labelled with the activity dependent marker c-fos after stepping in intact adult rats (Ahn et al., 2006, Tillakaratne et al., 2014) and elevating the treadmill incline (up to a 25° incline) significantly increased the number of such c-fos labelled central cluster cholinergic interneurons, some of which were located ventral or ventrolateral to the central canal (Tillakaratne et al., 2014). Although the specific function in the role of these central cluster cholinergic cells in the regulation of recruitment and modulation of specific motorneuron pools remains to be determined, these studies suggest an involvement in the adult rat during locomotion.

The lack of VAChT/GAD67-IR terminals in the ventral horn means the double labelled cells are unlikely to be the small set of cholinergic V0 interneurons (identifiable by expression of the paired like homeodomain transcription factor Pitx2) that represent the sole source of c bouton synapses onto motoneurons. Instead, they may correspond to central canal cluster cholinergic non-Pitx2 containing cells (Zagoraiou et al., 2009). However, the projections of these cells are not yet known. We did observe a small population of terminals containing both VAChT and GAD67-IR lateral, ventrolateral and dorsolateral to the central canal, presumably arising from the cells reported here. Identifying the projections and functions of these ChAT/GAD67-GFP cells should be facilitated through the use of double transgenic rats with ChAT and GAD cells labelled with different fluorescent markers (Saito et al., 2015) or intersectional genetics (Madisen et al., 2015) where only the ChAT/GAD67 cells are labelled, perhaps for opto- or chemo-genetic manipulation. Our finding that the GAD67-GFP/ChAT-IR population of lamina X cells is the same in juvenile and adult mice means that the typical use of juvenile mice for *in vitro* electrophysiology is a viable approach to investigate the functions of these cells.

Co-expression of GAD and ChAT is not unique to the spinal cord. Anatomical studies have revealed ChAT-IR neurons that also contained GAD and/or GABA-IR in the rat retina, cerebral cortex and basal forebrain (Kosaka et al., 1988), as well as the cat laterodorsal and pedunculopontine tegmental nuclei (Jia et al., 2003). ChAT-IR has also been observed in GAD67-GFP neurons in the mouse brainstem in the nucleus of the solitary tract, area postrema, reticular formation and lateral paragigantocellular nucleus (Gotts et al., 2015). In *in situ* hybridisation studies, mRNA for both GAD and ChAT is present in cell bodies of the globus pallidus and nucleus basalis of the mouse basal forebrain (Granger et al., 2016). GAD67/ChAT co-expression appears to be functionally relevant since paired patch clamp recordings in the rabbit retina revealed that ACh and GABA are co-released from starburst amacrine cells onto direction sensitive retinal ganglion cells (Lee et al., 2010). More recently, optogenetic studies have provided functional evidence of co-release of GABA and ACh from a population of globus pallidus cells projecting to the mouse cerebral cortex (Saunders et al., 2015a) and from neurons in the mouse forebrain (Saunders et al., 2015b). Furthermore, double transgenic rats, in which ChAT and vesicular GABA transporter express different fluorescent proteins revealed a population of acetylcholine and GABA producing neurons in the prepositus hypoglossi nucleus with unique electrophysiological and morphological properties (Saito et al., 2015). Similar studies using these strategies in rats to electrophysiologically record from ChAT/GAD cells in lamina X could therefore lead to an increased understanding of their functional phenotypes and indeed projections.

### 3.3 Conclusion

This study has revealed a population of ChAT-IR/GAD67-GFP neurons ventral to the central canal of the cervical, thoracic and lumbar spinal cord in the mouse, potentially corresponding to a sub-population of the cholinergic central canal cluster cells. To increase our understanding of the neuronal circuitry surrounding the central canal, the function of these cells needs to be determined. Our findings that juvenile and adult expression is similar enables such functional studies to be carried out in juvenile animals more commonly used for electrophysiology than adults.

## 4 Experimental procedure

### 4.1 Animals used

Adult (4–6 weeks, n=10) or juvenile (7–14 days, n=3) GAD67-GFP mice of either sex expressing GFP under control of the endogenous promoter for GAD67 (Tamamaki et al., 2003) or adult (4–6 weeks, n=4) wild type C57/BL6 mice were used in line with the Animals (Scientific Procedures) Act 1986 and the ethical standards set out by the University of Leeds Ethical Review Committee by individuals with UK Home Office approval. Every effort was made to minimise the number of animals used and their suffering.

### 4.2 Immunofluorescence for GAD67-GFP with either GAD67 or ChAT

GAD67-GFP adult mice were anaesthetised with sodium pentobarbitone (60 mg/kg) I.P. and perfused transcardially with either 2% glutaraldehdyde/4% paraformaldehyde (PFA) in 0.1 M phosphate buffer (for sections determining the distribution of GAD67-GFP with GAD67-IR) or 4% PFA (for sections determining the distribution of GAD67-GFP with ChAT-IR). The cervical, thoracic and lumbar sections of the spinal cords were dissected and post-fixed in 4% PFA overnight before washing in 0.1 M phosphate buffer and sectioned transversely at either 20 µm (for sections determining the distribution of GAD67-GFP with GAD67-IR) or 50 µm (for sections determining the distribution of GAD67-GFP with ChAT-IR) using a vibrating microtome (Leica VT1300S). Alternatively, some spinal cords were embedded in 10% gelatin, postfixed overnight in 1% glutaraldehyde in 4% PFA and 50 µm sagittal sections cut using a vibrating microtome. Sections were incubated in 1% bovine serum albumin (Molecular Probes) in PBS for thirty minutes, washed for 3×10 min in PBS and then simultaneously incubated with antibodies against GFP and GAD67 (diluted in 1% BSA in PBS, see Table 2) or GFP and ChAT (See Table 2) for 4 nights. Sections were then washed for 3×10 min in PBS.

GFP was detected by incubating the sections in AlexaFluor^488^ donkey anti rabbit (1:1000, Invitrogen) for 1 h. GAD67-IR was detected by incubating sections in biotinylated horse anti-mouse (1:200, Vector Labs) overnight at 4 °C, before washing in PBS and incubating in Streptavidin Alexa^555^ (1:1000, Invitrogen) for 1 h. ChAT-IR was detected by incubating sections in AlexaFluor^555^ donkey anti goat (1:1000, Invitrogen) for 1 h.

Sections were washed 3×10 min in PBS before mounting onto slides and covered with coverslips using Vectamount medium (Vector labs). A single plane image was taken under a confocal microscope (Zeiss LSM 510 Meta) in 3 sections at cervical, thoracic and lumbar levels for each animal and the degree of co-localisation of GAD67-GFP and either ChAT-IR or GAD67-IR in cell bodies noted.

### 4.3 Immunohistochemistry for vesicular acetylcholine transporter (VAChT) and GAD67

Transverse 50 µm sections of thoracic spinal cord were cut from 4 adult wild type C57/BL6 mice as above. To detect the distribution of vesicular acetylcholine transporter, sections were incubated in anti-VAChT antibody (see Table 2) overnight at 4 °C. The sections were washed 3×10 min in PBS before being incubated in AlexaFluor^555^ donkey anti goat (1:1000, Invitrogen) for 1 h. Sections were then incubated in anti-GAD67 antibody (see Table 2) overnight at 4 °C, washed for 3×10 min in PBS and then incubated in AlexaFluor^488^ donkey anti-mouse (1:1000, Invitrogen) for 1 h. Sections were washed in PBS and mounted on slides as above.

### 4.4 Microscopy and image capture

Sections were observed under either a Nikon Eclipse E600 microscope equipped with epifluoresence and images captured using a Q-Imaging Micropublishing 5.0 camera and Aquis image capture software or a confocal microscope (Zeiss LSM 510 Meta) and images captured using Zen software (Zeiss). Images were adjusted for brightness and contrast and compiled using CorelDraw x6 software. All confocal images shown are of single optical sections.

### 4.5 Quantification of ChAT-IR, GAD67-GFP containing neurons and dual labelled ChAT-IR/GAD67-GFP cells

ChAT-IR, GAD67-GFP and dual labelled ChAT-IR/GAD67-GFP containing cells in lamina X of the spinal cord were manually quantified from 3 adult and 3 juvenile mice. From each adult animal, every sixth consecutive serial section was studied and from each juvenile animal every third consecutive section was studied to avoid the risk of double counting the same neuron in adjacent sections. In adult mice 30 sections in total were studied; 10 sections each from cervical, thoracic and lumbar regions. In juvenile mice 15 sections in total were studied; 5 sections each from cervical, thoracic and lumbar regions. The numbers of cells were taken from each section and pooled. The width and length of the somata of labelled cells were measured using CorelDraw x6 software.

### 4.6 Reconstruction of the location of dual labelled ChAT-IR/GAD67-GFP neurons in lamina X of the thoracic spinal cord

The thoracic spinal cord (T1 to T12) of a GAD67-GFP adult mouse was sectioned sagittally and processed using immunohistochemistry for ChAT and GAD67-GFP as described above. The location of co-localised ChAT-IR and GAD67-GFP neurons in relation to the central canal region throughout the studied thoracic cord was observed and mapped using camera lucida. The location was then reconstructed using CorelDraw x6 software.

### 4.7 Data expression and nomenclature

All numerical data are expressed as mean values±standard error of the mean. N is used to represent the number of neurons counted.

## Figures and Tables

**Table 1:** The mean number of ChAT-IR, GAD67-GFP containing and dual labelled ChAT-IR/GAD67-GFP cells in lamina X of the spinal cord at the cervical, thoracic and lumbar regions in the adult and juvenile mouse.

	**Level of spinal cord (number of 50 µm sections counted)**	**Mean number of ChAT-IR cells per 50 µm section**	**Mean number of GAD67-GFP cells per 50 µm section**	**Mean number of ChAT-IR/GAD67-GFP dual labelled cells per 50 µm section**	**% of ChAT-IR cells that also contain GAD67-GFP**
**Lamina X of the adult mouse**	Cervical (30)	10.77±0.81	33.83±3.56	4.03±0.29	37%
Thoracic (30)	14.27±0.44	32.40±1.46	3.80±0.42	27%
Lumbar (30)	13.70±1.29	32.07±5.47	4.47±0.59	33%
**Lamina X of the juvenile mouse**	Cervical (15)	9.73±0.77	52±5.98	4±0.26	41%
Thoracic (15)	12.53±0.82	44±3.21	2.33±0.43	19%
Lumbar (15)	16.20±1.42	50.73±2.69	2.87±0.74	18%

**Table 2:** Primary antibodies used.

**Antibody**	**Immunogen**	**Manufacturer, catalog number, species raised against, type of antibody**	**Concentration of antibody (In PBS +0.1% Triton)**
ChAT (Choline acetyltransferase)	Human placental enzyme	Millipore Cat# AB144P raised in goat, polyclonal antibody	1:500
GFP (Green fluorescent protein)	GFP isolated directly from Aequorea victoria	Molecular Probes Cat# A11122, raised in rabbit, polyclonal antibody	1:1000
GAD67 (Glutamate decarboxylase 67)	Recombinant GAD67 protein	Millipore Cat# MAB5406, raised in mouse, monoclonal antibody	1:500
VAChT (Vesicular Acetylcholine Transporter)	KLH-conjugated linear peptide corresponding to the C-terminus of rat VAChT	Millipore Cat# ABN100, raised in goat, polyclonal antibody	1:1000
